# The Effect of Perceived Regional Accents on Individual Economic Behavior: A Lab Experiment on Linguistic Performance, Cognitive Ratings and Economic Decisions

**DOI:** 10.1371/journal.pone.0113475

**Published:** 2015-02-11

**Authors:** Stephan Heblich, Alfred Lameli, Gerhard Riener

**Affiliations:** 1 University of Bristol, CESifo, IZA and SERC, Department of Economics, Bristol, United Kingdom; 2 Research Centre *Deutscher Sprachatlas* and ADW Mainz, Marburg, Germany; 3 Düsseldorf Institute for Competition Economics, University of Mannheim and CRC PEG University of Göttingen, Mannheim, Germany; University Children’s Hospital Tuebingen, GERMANY

## Abstract

Does it matter if you speak with a regional accent? Speaking immediately reveals something of one’s own social and cultural identity, be it consciously or unconsciously. Perceiving accents involves not only reconstructing such imprints but also augmenting them with particular attitudes and stereotypes. Even though we know much about attitudes and stereotypes that are transmitted by, e.g. skin color, names or physical attractiveness, we do not yet have satisfactory answers how accent perception affects human behavior. How do people act in economically relevant contexts when they are confronted with regional accents? This paper reports a laboratory experiment where we address this question. Participants in our experiment conduct cognitive tests where they can choose to either cooperate or compete with a randomly matched male opponent identified only via his rendering of a standardized text in either a regional accent or standard accent. We find a strong connection between the linguistic performance and the cognitive rating of the opponent. When matched with an opponent who speaks the accent of the participant’s home region—the in-group opponent –, individuals tend to cooperate significantly more often. By contrast, they are more likely to compete when matched with an accent speaker from outside their home region, the out-group opponent. Our findings demonstrate, firstly, that the perception of an out-group accent leads not only to social discrimination but also influences economic decisions. Secondly, they suggest that this economic behavior is not necessarily attributable to the perception of a regional accent per se, but rather to the social rating of linguistic distance and the in-group/out-group perception it evokes.

## Introduction

Language as the primary means of human communication forms a large part of social practice. It is shaped by speakers’ idiosyncratic experiences [1] and by long-lasting cultural traits [2]. In everyday communication, both of these components, the individual and the cultural, evoke stereotypes and social ratings. Spoken language is thus a signal that elicits particular conceptualizations about the speaker [3]. The extent to which these determine non-linguistic behavior is still poorly understood.

The identifying potential of language has recently attracted researchers from different fields, including linguists [4,5], psychologists [6,7] and economists [8,9]. Measuring the effect of language on social behavior and individual interaction is however very difficult, given its dependence on specific contexts and individual preconditions. The most common way of addressing this challenge is to focus on individual attitudes and judgments. It turns out that listening to non-native or regional accents can invoke judgments about the credibility of speakers [7,10], their character traits and cognitive capacities [6,11], or even influence the perception of facts in criminal cases [12,13]. There is also evidence that the use of an accent can imply strategic advantages, e.g., in sales conversations [9] or job interviews [14], where accents suggest a joint identity. What remains open is to what extent attitudes really suit the action. While extensive research has already established a link between attitudes and action for individual characteristics like beauty or ethnicity [15–17], there has been widespread disregard for the gap between reported attitudes to accents and actual behavior. This is unfortunate, because accents are qualitatively different from other distinguishing factors (e.g., physical attractiveness) and effectively constitute a unique parameter of human interactions [18] that can be used strategically.

This paper addresses the gap between accent perception and individual action. Our first hypothesis is that accent perception does affect individual behavior. Since accents distinguish social groups, the effect should differ when perceiving in-group or out-group accents [19]. This is our second hypothesis. Finally, we take up the finding that out-group accents can invoke discriminatory judgments and pose our third hypothesis that in-group favoritism is manifested by a feeling of cognitive superiority.

To formally test these hypotheses, we combine techniques from experimental economics and linguistics to develop a picture of differentiated behavioral discrimination. To omit potential influences from cross-country differences we focus on regional accents within one country—in our case Germany—that indicate a higher similarity between speakers and listeners than foreign accents [14]. Regional accents typically originate in local dialects [20]. With the introduction of national radio and television programs later, dialects began to converge to the codified written language [21]. This process leaves us with regional accents as an intermediate stage between dialects and standard language today (note that other studies—especially in the German tradition—may use the terms ‘regiolect’, ‘regional dialect’, ‘regional standard’ or ‘spoken standard’ to refer to this intermediate stage). In Germany, regional accents typically consist of phonological and inflectional features that are still understandable for individuals from other regions. They are commonly used in everyday communication and subject to noticeable variation depending on contextual requirements [22,23]. Importantly, regional accents still reflect historic variation in norms, habits, and conventions that emerged over generations within dialect regions [24,25]. Already in childhood, regional accents turn out to be a more relevant dimension of social preferences than foreign accents or race [26,27]. This is why regional accents today still distinguish social groups that differ in acceptance, popularity and loyalty [28]. In the following, we explore this distinguishing feature of regional accents to assess how differences in accents affect individual interactions.

The main challenge for our research design is to account for the possibility that using an accent may either be strategic or correlated with context-specific and/or individual characteristics. Both cases would induce spurious correlations [29]. To overcome potential problems of confounding influences and identify an unbiased effect, our experiment meets the following three criteria. First, the controlled laboratory environment rules out any biases from unobserved context effects causing individuals to use regional accent strategically. This involves fixed interactions as well as fixed language treatments. Second, our experimental strategy separates accent effects from possibly confounding speaker characteristics like voice or intonation. Third, we present a strategy to distinguish general social discrimination of accent speakers from specific social discrimination of out-group accent speakers.

Our experimental setup confronts experimental participants (EP) with three types of randomly assigned language samples, one in German standard language (in the following *standard accent*) and two in regional accents. All language samples are provided by native language informants (LI). The first regional accent is chosen to match the Eastern Middle German accent spoken in the EPs home region, Thuringia. The second accent originates in a different region, namely Bavaria. Both accents rank among the most prominent accents in German census data [30]. Our setup implies that EPs perceive Eastern Middle German as *in-group accent* and Bavarian as *out-group accent*. The experiment consists of cognitive tests where EPs have to compare their own performance with the LI’s expected performance. If they expect to outperform the LI they can choose to compete. If successful, they will receive a higher remuneration but will lose money if their performance is equal or worse than the LI. If EPs do not rate their own performance higher than the LI’s performance, they can choose to cooperate instead, or choose a strategy that is independent of the LI’s performance. The only thing EPs know about their opponents is their (randomly matched) accent. Any systematic difference in the choice to cooperate or compete when being matched with an in-group or out-group LI thus reveals how accent perception affects action.

Our results confirm our first hypothesis, that accent perception affects individual interactions. Moreover, we find evidence for a systematically different treatment of the out-group accent. EPs are less willing to cooperate with the out-group accent LI and choose to compete instead. Since the spoken accent is not related to the LI’s performance by design, we follow [31] and consider this differential treatment as expression of social discrimination. To the extent that individuals can *choose* to use a standard accent or a regional accent, our results also allow for the reverse argument that individuals can influence the economic behavior of interaction partners through the choice to employ regional accents or standard accent.

In the remainder, we will explain our experimental setup in detail, present and discuss their relevance and implications.

## Material and Methods

### Experimental strategy

**Fig 1:**
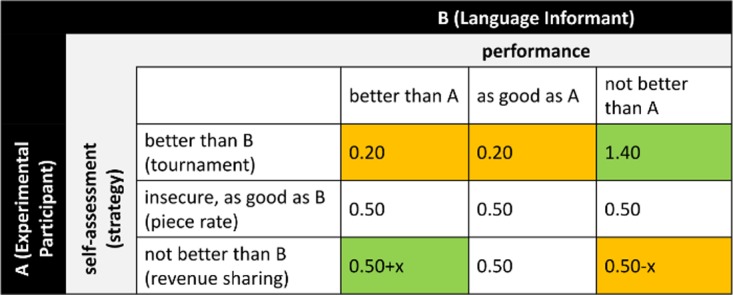
Experimental strategy and subsequent empirical analysis. The standard accent sample (*Stand*) is shown in white, the Bavarian accent (*Bav*) in gray, and the Thuringian accent (*Thur*) in black. The first stage of the experiment shows the two language informants (*LI*) who provide two language samples each. In the second stage, we relate economically relevant choices to the assigned treatments and match one of four language samples randomly with experimental participants (*EP*). In the analysis, we first estimate within-speaker differences to eliminate the effect of individual confounding characteristics (*First Differences*) and then calculate the difference in those first differences (*Second Difference*) to account for stochastic discrimination against regional accent. Contrasting the expected choices leaves us with an unbiased discrimination effect δ (cf. following explanations).

As outlined in Fig. 1 (upper part), our strategy proceeds in two stages. In the first stage, language informants (LI) with competence in standard accent and regional accent generate two language samples, one in standard accent and one in regional accent. Additionally, they perform a set of five cognitive tasks which are remunerated according to performance (piece rate).

In the second stage, we invite experimental participants (EP) to the laboratory where they perform the same set of cognitive tasks plus one extra task at the beginning. This first cognitive task involves one of the randomly assigned language samples generated in the first stage. EPs’ remuneration depends on their performance in these tasks as well. However, after the first task, EPs can also choose remuneration schemes that incorporate their expectation of the LI’s performance. If they believe that they are better than the LI they can compete, otherwise cooperate or ignore the LI. By linking the EPs’ choice to compete or cooperate to the randomly assigned language sample, we can assess how accent perception influences individual behavior.

### First Stage

**Language Informants (LI)**: All LIs were recruited at Marburg University. We invited candidates to a linguistic assessment, consisting of three tests. First, we tested the LI’s general competence in speaking standard accent without any regional marking. Secondly, we tested the LI’s competence in speaking with the regional accent of their home region using a competence test introduced by [32]. Thirdly, we tested their reading competence with both standard accent without regional marking and regional accent. To assess their performance, we use a dialectality measure developed by [33]. Based on the three tests, we chose one LI from Bavaria (originating from Ingolstadt) and one LI from Thuringia (originating from Erfurt). Both LIs were socialized in regional accent and standard accent and speak it interchangeably. Both were 23 year old male undergraduate students.

**Language Samples**: We generated four language samples that represent our language treatments. Each LI provided two language samples, one in standard German and one in either the in-group accent (Thuringian) or the out-group accent (Bavarian). Employing the same LI for a language sample in a regional accent *and* one in a standard accent provides a constant voice quality that allows us to account for confounding individual characteristics like tone pitch, speech rate or intonation. At the same time this enables us to control social characteristics such as age or sex [34]. Similarly, we focused on male speakers to avoid gender effects. Finally, we acoustically normalized the speech signals so that our language samples were played back at the same volume, eliminating noise from breathing or throat clearing, and standardizing the time signals (pauses).

The language samples comprised of an 81 word accident report and a list of words. The accident report was designed to include language features that characterize both dialects. For the Thuringian sample this included, e.g., the centralization of back vowels [u:] and [o:] or the weakening of unvoiced consonants [p] and [t] of voiced consonants [b] and [d]. For the Bavarian sample this included replacing rounded front vowels [y:] and [ø:] by unrounded [i:] and [e:] or using the apical instead of the uvular /r/. The standard accent sample has no such regional phenomena. All samples lasted approximately 30 seconds.

**Fig 2:**
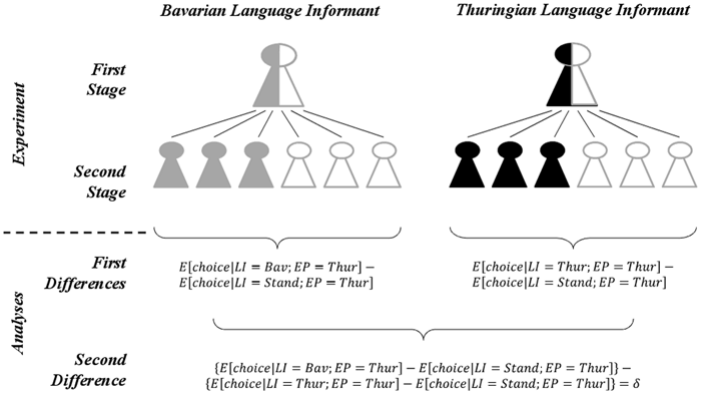
Regional intensity across the language samples. The figure shows the regional intensity of our language samples relative to codified standard German (d = 0). We find a strong and comparable deviation of both regional language samples from codified standard. At the same time, we find an insignificant difference between these two regional accent samples and between the two standard accent samples.

Fig. 2 shows the results of a formal linguistic test [33,35] comparing the regional intensity of our language samples. The intensity d is calculated as the number of micro-phonetic features (e.g. voicing, manner, or location of articulation) that deviate from codified standard German divided by the overall number of words in the text. A value of d = 0 would suggest perfect compliance with codified standard German. A value of d = 1 means that, on average, one phonetic feature per word differs from codified standard German. Very pronounced local accents may have a score of d > 2 or even d > 3 [33,22]. [36] finds that an intensity of d > 2 leads to intelligibility problems. Reassuringly, we do not measure an intensity larger than d = 2 in our language samples. We further find that neither the two interregional accent samples (Bavarian vs. Thuringian: T = -1.079, p = .284) nor the standard accent samples (Bavarian vs. Thuringian: T = -1.354, p = .180) show a statistically significant difference while the individual speakers’ samples in the regional accent and the standard accent are significantly different; (regional accent vs. standard accent: Bavarian: T = 6.801, p <. 001; Thuringian: T = 8.545, p <. 001). The latter nicely illustrates the dual competence of our LIs. At the same time it is reassuring that our design rules out biases from differing accent intensities.

### Second Stage

**Experimental Participants (EP)**: The second stage of the experiment was conducted at the computer laboratory at the University of Jena in September 2010 and September 2011. EPs were recruited via the ORSEE online recruitment system [37]. Two selection criteria were involved in the choice of EPs: (i) we excluded economics and linguistics students and (ii) we excluded persons who had already performed similar tasks in a related experiment. Overall, we conducted 19 laboratory sessions with 18 EPs per session, leaving us with a total of 342 observations.

Our research design required that we focus on EPs who originated from the same dialect region. Unfortunately, ORSEE does not include information about the participants’ home region. Instead, we had to select the relevant observations after the experiment, based on a questionnaire, following two criteria. Firstly, we chose EPs from the federal states of Thuringia and Saxony, which comprise the Eastern Middle German dialect group. Secondly, we only selected EPs who had grown up in this dialect region and whose parents also came from there. This latter criterion ensures that the EPs share a comparable social background. After dropping all EPs that did not meet these criteria, we ended up with a subset of 167 EPs who were primarily undergraduate students at the University of Jena.

Within each session we randomly matched the four language samples provided by the LIs in the first stage with the EPs. This procedure avoids confounding treatment effects with session effects. Moreover, EPs were not aware of the other treatments, so as to reduce experimenter demand effects (cf. [38]). We obtained the following observation numbers for each pairing of EP and LI: standard accent spoken by the Thuringian LI: N = 41; standard accent spoken by the Bavarian LI: N = 40; Thuringian accent: N = 36; Bavarian accent: N = 50.

**Tasks**: The experiment consisted of eight sets of tasks (cf. Table 1), all of them programmed in zTree [39]. Tasks 1–5 were designed as cognitive tests that have been shown to be good predictors of general economic success (cf. Supporting Information A in S1 Supporting Information for examples of these tasks). They cover a wide range of cognitive abilities including (a) language competence (tasks 1, 4), (b) the ability to abstract (task 2), (c) logic (task 3), and (d) memory (task 5);skills that have been shown to be important determinants for success in the labor market [40]. To control for individual preferences that may affect the choice of a payment scheme we further included standard games that test tournament and risk aversion (tasks 6–7). A final task presented a questionnaire that helps us collected personal and linguistic information about the EPs (task 8).

**Table 1:** Procedure of the experiment.

		Stage 1	Stage 2
Task		Language Informants (LI)	Experimental Participants (EP)
1	Listening Comprehension		X
**2**	**Mathematics**	**X**	**X**
**3**	**Logic**	**X**	**X**
**4**	**Language**	**X**	**X**
**5**	**Memory**	**X**	**X**
6	Tournament Aversion		X
7	Risk Aversion		X
8	Questionnaire		X

Tasks 2–5 (bold) are the four experiments that are performed by LIs in stage 1. Tasks 1–8 are subsequently performed by the EPs in stage 2. In this set of tasks, EPs can choose their degree of interaction with the LIs.

*Task 1—Listening Comprehension*: The regional treatments were introduced in a listening comprehension task at the beginning of the experiment. In this task, EPs listened individually on headphones to a text read out by a randomly matched LI who spoke either standard German, or with a Bavarian or Thuringian accent. This 81-word accident report took a format familiar from radio news bulletins. After listening to the text, EPs were asked to answer multiple choice questions about it. This setting was designed as a typical listening comprehension task as encountered in school or university language courses. The text we used is part of the German language examination (*Deutsche Sprachprüfung für den Hochschulzugang*; DSH) required for university entrance in Germany. In all setups, EPs had to remember a number of facts from the text. In this context, the use of regional accents presented an additional test complication.

*Task 2—Mathematics*: The mathematics task required EPs to add up five two-digit numbers. Calculators were not allowed but paper and pencil were provided by the experimenter. All numbers were randomly drawn and presented in the following way: An open-ended series of calculations was to be performed within a set time of five minutes. As soon as EPs had completed one task they received a new one. A count of correctly solved calculations was always visible on the screen. After receiving the instructions, EPs could familiarize themselves with the task in a two-minute non-paid trial round. This task was included on the basis of [41] studies of male and female attitudes towards competition.

*Task 3—Logic*: In this task, EPs were asked to answer questions from the 2002 GRE (Graduate Record Examination) logic section. GRE is a standardized test used as a recruitment tool for doctoral candidates in Europe and the US. Each question described a particular situation on which the EPs had to answer several combinational logic-based questions.

*Task 4—Language*: In the language task, EPs were asked to place five words in order into a grammatically correct sentence (declarative sentence). Each word was assigned a unique number and EPs had to order these numbers to specify the correct sentence structure. Whenever they completed one sentence correctly, a new one was presented until five minutes had passed. This task had previously been used to analyze gender task stereotypes [42].

*Task 5—Memory*: As in the listening comprehension task EPs listened to a list of 16 words read out by the LI in a treatment-specific variety (i.e., either in standard accent or regional accent). The EPs were asked to memorize as many words as possible. Subsequently, they were presented with a list of words on the screen and the EPs had to identify the words that had previously been read out.

*Task 6—Tournament Aversion*: To test for tournament aversion, we used the results from the word order task (which is according to [42] only mildly gender-biased) and asked EPs whether they wanted to receive € 1 or to have their result compared with a randomly chosen result from another individual in the room. If their result was better, they would receive € 3, if not, they would forfeit payment.

*Task 7—Risk Aversion*: To control for risk aversion, we applied a simplified procedure based on [43] that had previously been used by [44]. EPs were presented with a list of five pairs of different lotteries. In each case, EPs had the choice between a safe lottery X that guaranteed payment of € 0.50 and a risky lottery Y in which they had an equal chance of winning amounts ranging from € 0.90 to € 1.50 or zero. In general, we would expect more risk adverse individuals to be slower to switch from lottery X to lottery Y. One pair of lotteries was randomly selected and the decision was paid out.

*Task 8—Questionnaire*: At the end, EPs were asked to fill out a questionnaire. This included biographic information and four sets of linguistic questions that helped us determine the EPs’ attitudes toward dialects and their region of origin.

**Payment Schemes**: In stage one of our experiment, LIs had been asked to perform the same four tasks that the EPs were asked to do in the laboratory. LIs earned a piece rate of one ECU (Experimental Currency Unit) per correct answer. The exchange rate between ECU and Euro is 1 EUR = 1.7 ECU. This exchange rate is calculated from a pilot study and provides that students earn on average the hourly wage rate of a research assistant at the University of Jena.

**Fig 3:**
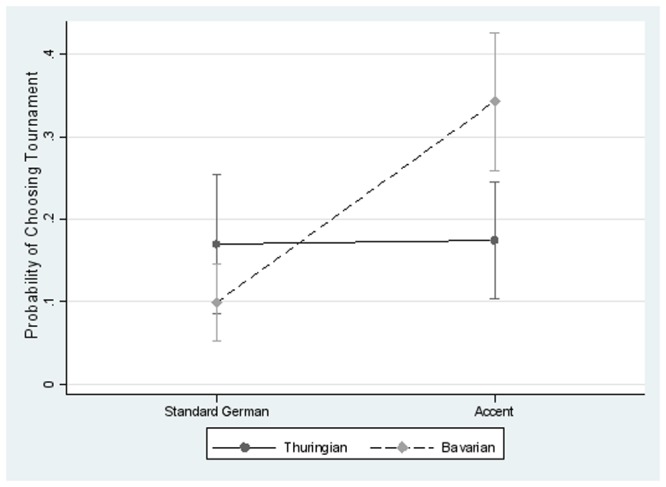
Payoff matrix for tasks 2–5. Color indicates potential gains (green) and losses (orange) compared to piece rate. Piece rate: m = Ʃc_a_ * € 0.50; revenue sharing: m = (Ʃc_a_ + Ʃc_b_) / 2 * € 0.50; tournament: m = Ʃc_a_ * € 1.40 if Ʃc_a_ > Ʃc_b_ otherwise m = Ʃc_a_ * € 0.20, with m = payoff; c_a_ = successful completion of task by participant A; c_b_ = successful completion of task by speaker B.

In stage two, EPs received € 0.50 per correct answer in Task 1 as an incentive to listen carefully. After that, EPs were informed that they would subsequently perform four other tasks that their matched LI had completed earlier for a fixed remuneration per correctly solved task. Tasks 2–5 were remunerated according to one of the following payment schemes (cf. also Fig. 3).

1. *Piece rate*: EPs were paid € 0.50 for each correctly solved piece independent of other subjects’ performance.
2. *Tournament*: The EP’s score in a specific task fis compared to the matched LI‘s score. If the EP’s score was higher than the LI‘s score; she earned €1.40 per piece. If they were equal to or lower; they received € 0.20 per piece.
3. *Revenue sharing*: EPs’ earnings were based on the average score of the EP and the LI, and paid at a piece rate of € 0.50.

EPs chose a payment scheme for tasks 2–5 *before* performing a task. In task 5 (memory task); they were not informed that the list was to be read out by the matched LI. The remuneration schemes suggest that EPs would choose tournament if they thought they were better than the LI and revenue sharing if they were afraid to score less. Piece rate is the outside option in case EPs do not want to compare themselves to anybody. Since the language sample was the LI’s only known characteristic, any systematic difference in the EPs choice to cooperate or compete was caused by their accent perception. Specifically, the EPs’ choice helps us uncover whether out-group LIs are more often found in a competitive situation. We would interpret a significantly lower willingness to cooperate as an indication of social discrimination because speaking with a regional accent is independent of the LI’s performance in the tasks.

The average experimental session lasted approximately 75 minutes and EPs earned an average payoff of € 10.13. This payoff is roughly equivalent to the hourly wage of a research assistant at German universities. The maximum (minimum) payoff amounted to € 31.10 (€ 3.40, respectively).

### Empirical Strategy

We analyzed the experimental outcome in a difference-in-differences framework (cf. Fig. 1; lower part). The first differences compare the choices of EPs who are matched with the same LI speaking regional accents (*Bav*/*Thur*) or standard accent (*Stand*). Differentiating between the expected choice of those EPs who were listening to the LI speaking Thuringian (Bavarian) accent and those who were listening to the same informant speaking with the standard accent reveals EP’s judgment of regional accents relative to standard accent. Since we are comparing the same LI in two contexts (regional accents and standard accent) this measure is independent of all fixed characteristics of the LI (like tone pitch or intonation) that may systematically affect the EPs’ choice.

These first differences are then compared with each other to measure the discrimination against the out-group regional accent relative to the in-group regional accent. The second difference (δ) thus measures whether EPs choose tournament significantly more often when being matched with the out-group LI. In this way, we can distinguish between general social discrimination of accent speakers and a specific social discrimination of the out-group accent speaker.

Empirically, this setup corresponds to a simple dummy variable interaction model with two main effects for language (regional accent or standard accent) and origin (Thuringia and Bavarian) and an interaction term that marks observations matched with a Bavarian accent. To calculate this difference-in-differences, we employ the following model to estimate EPs’ choice in the four different tasks:

$$scheme_{i}=\alpha +\beta_{1}Bavarian_{i}+\beta_{2}Accent_{i}+\beta_{3}Bavarian_{i}\times Accent_{i}+{X^{’}}_{i}y+\epsilon_{i}$$ 1

where *scheme* is a categorical outcome variable describing the payment scheme chosen by EP indexed *i*. *Bavarian* is an indicator variable taking the value 1 if the LI is from Bavaria and 0 if he is from Thuringia. Similarly, *Accent* is an indicator variable taking the value 1 if the language sample is regional accent (Thuringian or Bavarian) and 0 if it is standard accent. *Bavarian* × *Accent* is the interaction between the two indicator variables *Bavarian* and *Accent*. Although the inclusion of control variables is not necessary as the treatments are orthogonal to any other influencing variable, the controls may improve the precision of the estimates obtained and help us to identify the sources of observed discrimination patterns. Accordingly, *X*_*i*_ stands for a matrix of individual-level control variables that might influence the choice of the payment scheme including session fixed effects, age, gender, self-assessed performance, and the results from task 6 and task 7 indicating tournament aversion and risk aversion. Finally, ɛ is an *i*.*i*.*d*. error term.

The two parameters of interest are *β*_*2*_ and *β*_*3*_. *β*_*2*_ gives us the probability of choosing competition when the Thuringian LI speaks with a regional accent and *β*_*3*_ gives us the change in probability of choosing competition when the Bavarian LI speaks with a regional accent.

## Results

Table 2 summarizes the experimental design in the second stage. While there are no major differences in the scheme choice between the Thuringian and the Bavarian LI in the standard accent condition, there is a large increase of over 12 percentage points between the rates of choice of tournament when the Bavarian rather than the Thuringian accent is perceived from the same LI (Table 2).

**Table 2:** Summary statistics of payment scheme choice pooled over all tasks.

	Standard Accent		Regional Accent	
Origin of speaker	Thuringia	Bavaria	Thuringia	Bavaria
Revenue sharing	29	41	37	52
	17.68%	25.62%	25.69%	26.00%
Tournament	35	27	27	62
	21.34%	16.88%	18.75%	31.00%
Piece rate	100	92	80	86
	60.98%	57.50%	55.56%	43.00%

Number of observations	164	160	144	200
Pearson χ2	0.185	0.185	0.022	0.022
Fisher’s exact	0.185	0.185	0.021	0.021

Table 2 shows the number of times EPs chose each payment scheme by accent and LI origin pooled over all tasks.

Comparing the distributions of scheme choices for standard accent or regional accent spoken by the Bavarian LI or the Thuringian LI, there is a significant difference only in the regional treatments. This is confirmed by a multinomial logit model. EPs do not choose the tournament more often when perceiving the Thuringian accent, but there is a significant increase in tournament take up when matched with the Bavarian accent over all tasks (Pearson’s χ² <. 05).

**Fig 4:**
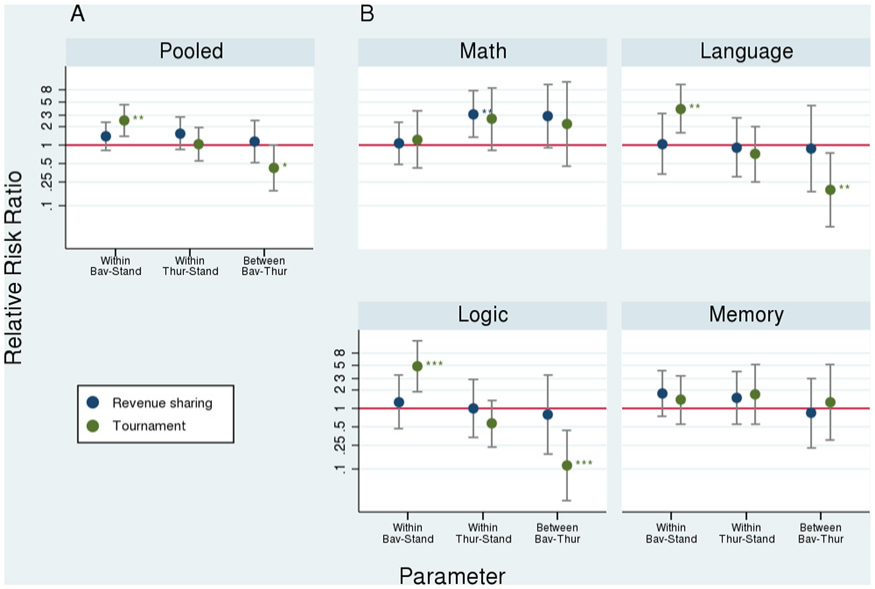
Predicted margins of Bavarian accent conditional on age and gender controls with the 90% confidence interval. Standard errors are clustered by subject. This graph shows the predicted margins of the probability of choosing tournament from a multinomial logit model (Table 3) using age and gender as additional control variables. EPs do not chose the tournament more often when perceiving the Thuringian accent, but tournament take up increases strongly when perceiving the Bavarian accent.

Fig. 4 summarizes the predicted margins of tournament take-up, showing a significant impact of Bavarian accent on tournament.

**Table 3:** Multinomial logit model of scheme choice.

	(1)		(2)		(3)		(4)		(5)	
	All		Math		Logic		Language		Memory	
	RS	Tourn	RS	Tourn	RS	Tourn	RS	Tourn	RS	Tourn
Constant	-1.586**	-2.425***	-2.905**	-3.960**	-4.405***	-3.315**	-0.709	-1.619	-1.795	-1.803
	(0.638)	(0.747)	(1.440)	(1.893)	(1.549)	(1.643)	(1.619)	(1.224)	(1.196)	(1.434)
Accent: Thuringian	0.438	0.088	1.122**	1.060	-0.109	-0.263	0.003	-0.512	0.337	0.535
	(0.304)	(0.316)	(0.541)	(0.764)	(0.662)	(0.688)	(0.673)	(0.546)	(0.602)	(0.686)
Standard German: Bavarian	0.453	-0.255	1.096**	0.884	-0.161	-0.364	-0.203	-1.826***	0.689	0.853
	(0.297)	(0.306)	(0.521)	(0.711)	(0.673)	(0.637)	(0.644)	(0.618)	(0.582)	(0.636)
Bavarian X Accent	-0.150	0.883**	-1.084	-0.847	0.115	1.748*	0.248	2.258***	0.182	-0.228
	(0.408)	(0.426)	(0.728)	(0.995)	(0.961)	(0.896)	(0.908)	(0.815)	(0.796)	(0.879)
Logic	-1.491***	-0.030								
	(0.292)	(0.306)								
Language	-1.200***	0.478								
	(0.287)	(0.298)								
Memory	-0.766***	-0.105								
	(0.259)	(0.316)								
Age	0.049**	0.019	0.096*	0.066	0.110**	0.026	-0.036	0.004	0.036	0.012
	(0.022)	(0.026)	(0.055)	(0.070)	(0.054)	(0.060)	(0.061)	(0.043)	(0.042)	(0.051)
Envious	-0.205	0.824***	-0.272	0.907*	-0.059	1.263***	0.110	1.144***	-0.478	0.101
	(0.222)	(0.212)	(0.389)	(0.500)	(0.541)	(0.434)	(0.502)	(0.399)	(0.424)	(0.434)
Female	0.421**	-0.805***	0.271	-1.643***	0.721	-0.973**	0.374	-0.301	0.340	-0.785*
	(0.214)	(0.215)	(0.379)	(0.568)	(0.536)	(0.440)	(0.481)	(0.394)	(0.409)	(0.444)
Observations	656	656	164	164	164	164	164	164	164	164

To assess the statistical significance of the observation in Table 2 while controlling for session effects and socio-economic characteristics, we estimate the multinomial logit model outlined in Equation 1, adding the variable *Envious* as control, in order to control for a general tendency to prefer tournaments. We observe that EPs do not chose the tournament more often when perceiving the Thuringian accent, but there is a significant increase in tournament take up over all tasks when EPs perceive the Bavarian accent. Standard errors in parentheses (clustered on the individual level in model (1)). Multinomial logit model, dependent variable Choice of payment scheme;

*p <. 10,

**p <. 05,

***p <. 001

When the outcome categories of revenue sharing and piece rate are pooled, in the aggregate (Fig. 5; A) EPs choose tournament more often when they perceive the Bavarian accent (robustness is also given when estimating a mixed model, cf. Table A in S1 Supporting Information). This result is largely driven by two tasks (Fig. 5; B): the logic task (rr_Tourn/Bav_ = 3.88, p <. 05), which is particularly aimed at problem solving skills, and the language task (rr_Tourn/Bav_ = 4.84, p <. 01), which targets linguistic performance. At the same time, there is some indication that EPs avoid the tournament option when listening to the Thuringian accent in these tasks. In the math task (ability to abstract) and the memory task (ability to store and recall information) we do not find such behavior. One explanation for the effect heterogeneity is that EPs presume that the performance in the language and logic tasks depends on linguistic abilities. Our results then show that EPs expect the out-group accent speaker to perform worse but not the in-group accent speaker. The assumption that out-group accent speaker have less linguistic abilities is a clear indication of region-specific clichés and stereotypes.

Fig. 5. Relative risk ratios (*rr*; log scaled). *Pooled* refers to the joint results for all tasks using clustered standard errors at the individual level; the remaining graphs present results per task. *Within* refers to the Analyses—First Differences stage in Fig. 1 (within-speaker differences) and *Between* to the Analyses—Second Difference stage (differences between speakers). A rr of one (e.g., Within Bav-Stand; Tournament) would indicate an equal likelihood that EPs choose tournament whether listening to Bavarian accent (*Bav*) or standard accent (*Stand*) while, e.g., a rr of 2.46 would indicate that EPs were 2.46 times more likely to choose tournament when listening to the Bavarian accent than when the same LI were to speak standard accent; A rr of less than one (e.g., Within Thur-Stand; Tournament) indicates that EPs avoid tournament when listening to the Thuringian accent (*Thur*) more often than in the standard accent treatment (*Stand*).

The difference between the two regional accents also pertains when controlling for the guessed rank of the EPs. EPs choose tournament more often when they thought they were better than their opponent, but this does not change the size or the significance of the treatment variables.

To control for individual preferences that might affect the choice of the payment scheme, we elicited social preferences regarding the opponent and furthermore controlled for tournament aversion (task 6) and risk aversion (task 7). In all cases the results remain stable. EPs who indicated tournament aversion tend to opt for competition less often while envious people are more likely to engage in tournament. Moreover, there are no interaction effects between the accent treatments and gender, indicating that men and women exhibit the same behavior.

Finally, EPs were asked to fill in a questionnaire (task 8) that provides additional information about their personal biographies and attitudes towards language. These results allowed us to capture the EPs’ behavior more precisely. Using 36 validated rating scales from [45], we calculated a measure of linguistic loyalty by aggregating EPs’ attitudes towards the use of regional language varieties in everyday contexts (e.g., school or family. Further details on the measure along with the individual items of all rating scales are provided in the Supporting Information, cf. Supporting Information B and Fig. A in S1 Supporting Information). This measure allowed us to distinguish N = 134 EPs who show a high loyalty to regional varieties (Table B; Panel A). The results illustrate the complementarity of experiments and questionnaires in exploring the link between economically relevant behavior and stated attitudes. Those EPs who are classified as being loyal to regional varieties are not equally loyal to all regional accents. On the one hand, they are loyal to the in-group regional accent. Matched with the Thuringian accent sample, they avoid tournament significantly more often (rr = .364, p <. 05) thus indicating a similarity-attraction effect. On the other hand, they choose tournament significantly more often (rr = 3.453, p <. 001) when confronted with the distant Bavarian accent.

## Conclusion

Our experimental approach allows us to evaluate accent discrimination in economically relevant situations. Each of the tasks performed in the experiment addresses different cognitive capacities. Our experimental setup requires EPs to then compare their own capacities with the LIs’ expected cognitive capacities. EPs reveal the result of this comparison through their choice of a payment scheme. If they believe that they can outperform the LI they choose competition over cooperation.

Varying the accent exogenously, we find that the economic behavior of the interaction partners is undoubtedly influenced by the use of regional accent or standard accent (first hypothesis). However, it turns out that only the out-group regional accent affects economic behavior, indicating that regional accents also transport socio-symbolic information about the speaker [46]. This is a clear pattern of in-group vs. out-group behavior (second hypothesis)). Since EPs are more likely to try and outcompete the opponent (i.e. choose competition) when being matched with the distance accent LI, we interpret this as feeling of cognitive superiority against the out-group speaker (third hypothesis).

Looking at the four tasks separately, we observe effect heterogeneity related to the cognitive dimensions tested in the experiment. In the language and logic tasks, EPs are more likely to choose tournament against the out-group accent LI (Bavarian). They think they are better. When being matched with the in-group accent LI (Thuringian) we do not find this behavior. Beyond that, we find weak indication that EPs are more likely to cooperate (i.e. choose revenue sharing) with the in-group accent LI in the math task and we do not find any effect in the memory task.

One explanation for the effect heterogeneity is that EPs consciously or subconsciously relate language and combination tasks, but not math and memorization tasks, to the language treatment. In that case, regional clichés and stereotypes about the out-group accent would drive the EPs’ expectation about the opponent’s performance in language related tasks. Another potential explanation may relate to the EPs’ familiarity with the type of task. While mental arithmetic and memorization are explicitly trained from early on in kindergarten and at school, cognitive tests (so called brain teasers that are commonly used in assessment centers) require combinational logic that appears only later in the school curriculum—if at all. To the extent that (i) everyone received training in math and memorization at school and (ii) almost everyone has had the experience that sometimes somebody else was better in solving these tasks, it may be rational to avoid competition. By contrast, language and combinational logic tasks are not explicitly trained at school thus leaving more room for assumptions about the opponent’s performance. This is where regional clichés and stereotypes come into play. This interpretation relates to [47] who find that while trained persons tend to underestimate their performance, untrained persons tend to overrate their performance and ability. In our context, this may also explain why experimental participants tend to cooperate with the speaker of their own regional accent in the math task—thus looking for trustful support—while they don’t when the same person speaks standard language.

Since our results do not allow us to further explore these potential explanations, we refer them to future research. Based on our findings, we conclude that regional accents can encourage clichés and stereotypes that affect individual behavior. More generally, this carries implications for both, speakers and listeners. From the perspective of the listener, stereotypes may be goal-oriented [48] as they help reach a decision. But they also may be misleading and thus not economically effective. This opens up an interesting perspective for the speaker: the use of regional accents can be strategically employed to persuade or manipulate a communication partner.

## Supporting Information

S1 Supporting Information Supporting Information file. *Supporting Information A*: Material for replicating the tasks. *Supporting Information B*: Loyalty Measure. *Fig*. *A*: Multi-dimensional plot resulting from the exploration of linguistic loyalty using the questionnaire from (*29*). *Each* of the statements below (English translation) was rated on a seven step scale between the poles “completely agree” and “strongly disagree”. Black circles (cluster 1) = statements on dialects with negative connotations (e.g., “Dialect is vulgar.”), black triangles (cluster 4) = statements on dialects with positive connotations (e.g., “Dialect conveys a feeling of security.”), white triangles (cluster 2) = statements on standard German with positive connotations (e.g., “Standard German sounds elegant.”), white circles (cluster 3) = statements on standard German with negative connotations (e.g., “Standard German sounds stiff.”). Kruskal’s test = .081. *Fig*. *B*: Dialect intensity across the speech samples. Fig. A shows the results of a formal linguistic test comparing the dialect intensity (i.e. regional accent in the given case) of our speech samples relative to standard language. The figure shows a strong and comparable deviation of both regional speech samples from standard language; the standard German samples are comparable with almost no indication of dialect features. The latter nicely illustrates the dual competence of our language informants. At the same time, we measure a small and insignificant difference in the dialect intensity between the two regional accent samples and between the two standard language samples suggesting that the difference does not influence language perception or affect the semantics of the text (*32*, *33*). We calculate distance as number of micro-phonetic features like voicing, manner, or location of articulation that deviate from standard language divided by the overall number of words in the text (*22*). A value of d = 0 would suggest perfect compliance with standard language; a value of d = 1 means that, on average, one phonetically feature per word differs from standard language; very pronounced local dialects may have a score of d > 2 or even d > 3 (*22*, *32*, *33*). The difference between the regional accent samples is significant at p <. 001, the difference between the samples of the spoken standard language is not significant (cf. main text). *Table A*: The table shows panel regressions of tournament take-up where the outcome categories of revenue sharing and piece rate are pooled. *Column 1* presents the results from a random effects panel regression on the choice of tournament and *column 2–4* present mixed models. In *column 3* we additionally control for the guessed rank of the EPs. Finally, in *column 4* we add a full set of controls. Standard errors in parentheses; *p <. 10, **p <. 05, ***p <. 001. *Table B*: Linguistic loyalty and payment regime choice: Splitting the sample by loyalty measures, we observe that EPs with a high dialect loyalty (Panel A) choose tournament significantly more often when perceiving the distant Bavarian accent and significantly less often when perceiving the Thuringian accent. The same holds for EPs with standard German loyalty (Panel B), though the effect is less pronounced than it is in the case of dialect loyalty. For those EPs who have a low loyalty for dialects or standard German we do not find any effects. We report relative risk ratios; t statistics in parentheses. *p <. 05, **p <. 01 ***p <. 001
(DOCX)

## References

[1] Krauss RM, Chiu C (1997) Language and social behavior In: Gilbert D, Fiske ST, Lindsey G, editors. Handbook of Social Psychology. Boston: McGraw-Hill pp. 41–88.

[2] Cavalli-Sforza LL (2000) Genes, peoples and languages. London: Allen Lane 227 p.

[3] Ramus F, Hauser MD, Miller C, Morris D, Mehler J (2000) Language discrimination by human newborns and by cotton-top tamarin monkeys. Science 288: 349–351. 10764650 10.1126/science.288.5464.349

[4] de Fina A (2007) Code-Switching and the Construction of Ethnic Identity in a Community of Practice. Language in Society (LSoc) 36 (3): 371–392.

[5] Preston D (2010) Mapping the geolinguistic spaces of the brain In: Lameli A, Kehrein R, Rabanus S, editors. Language and Space. An International Handbook of Linguistic Variation. Berlin, New York: Mouton de Gruyter.

[6] Rakić T, Steffens MC, Mummendey A (2011) When it matters how you pronounce it: The influence of regional accents on job interview outcome. British Journal of Psychology 102: 868–883. 10.1111/j.2044-8295.2011.02051.x 21988389

[7] Lev-Ari S, Keysar B (2010) Why don’t we believe non-native speakers? The influence of accent on credibility. Journal of Experimental Social Psychology 46: 1093–1096.

[8] Ginsburgh V, Weber S (2011) How many languages do we need? The economics of linguistic diversity. Princeton: Princeton University Press.

[9] Mai R, Hoffmann S (2011) Four positive effects of a salesperson’s regional dialect in services selling. Journal of Service Research 14: 460–474.

[10] Tsalikis J, Ortiz-Buonafina M, LaTour MS (1992) The Role of Accent on the Credibility and Effectiveness of the International Business Person: The Case of Guatemala. International Marketing Review 9 (4): 57–72.

[11] Holmes K, Murachver T, Bayard D (2001) Accent, appearance and ethnic stereotypes in New Zealand. New Zealand Journal of Psychology 30 (2): 79–86.

[12] Frumkin L (2007) Influences of accent and ethnic background on perceptions of eyewitness. Psychology, Crime and Law 13: 317–331.

[13] Dixon JA, Mahoney B, Cocks R (2002) Accents of guilt? Effects of regional accent, ‘race’ and crime type on attributions of guilt. Journal of Language and Social Psychology 21: 162–168.

[14] Deprez-Sims AS, Morris SB (2010) Accents in the workplace: their effects during a job interview. International Journal of Psychology 45 (6): 417–426. 10.1080/00207594.2010.499950 22044081

[15] Hamermesh DS, Biddle JE (1994) Beauty and the labor market. The American Economic Review 84: 1174–1194.

[16] Purnell T, Idsardi W, Baugh J (1999) Perceptual and phonetic experiments on American English dialect ientification. Journal of Language and Social Psychology 18: 10–30.

[17] Mobius MM, Rosenblat TS (2006) Why beauty matters. The American Economic Review 96: 222–235.

[18] Gluszek A, Dovidio JF (2010) The way they speak: A social psychological perspective on the stigma of nonnative accents in communication. Personality and Social Psychology Review 14: 214–237. 10.1177/1088868309359288 20220208

[19] Romaine S (1980) Stylistic Variation and Evaluative Reactions To Speech: Problems in the Investigation of Linguistic Attitudes in Scotland. Language and Speech 23 (3): 213–232.

[20] Chambers JK, Trudgill P (1999) Dialectology. Cambridge: Cambridge University Press.

[21] Auer P (2005) Europe’s Sociolinguistic Unity, or: A Typology of European Dialect / Standard Constellations In: Delbecque N, van der Auwera J, Geeraerts D, editors. Perspectives on Variation. Berlin: Mouton de Gruyter pp. 7–42.

[22] Kehrein R (2012) Regionalsprachliche Spektren im Raum Zur linguistischen Struktur der Vertikale. Stuttgart: Steiner 389 p.

[23] Schmidt JE Formation of and change in regiolects and (regional) dialects in German. Taal & Tongval 63, 1 10.1002/acr.22531 25504789

[24] Coupland N, Bishop H (2007) Ideologised values for British accents. Journal of Sociolinguistics 11: 74–93.

[25] Falck O, Heblich S, Lameli A, Südekum J (2012) Dialects, cultural identity, and economic exchange. Journal of urban economics 72: 225–239.

[26] Souza AL, Byers-Heinlein K, Poulin-Dubois D (2013) Bilingual and monolingual children prefer native-accented speakers. Frontiers in Psychology 4 (953). 10.3389/fpsyg.2013.00953 24391616 PMC3870285

[27] Kinzler KD, Dupoux E, Spelke ES (2007) The native language of social cognition. Proceedings of the National Academy of Science 104 (30): 12577–12580. 17640881 10.1073/pnas.0705345104 PMC1941511

[28] Thorne S (2005) Accent pride and prejudice: Are speakers of stigmatized variants really less loyal. Journal of Quantitative Linguistics 12: 151–166.

[29] Manski CF (1993) Identification of endogenous social effects: The reflection problem. The Review of Economic Studies 60: 531–542.

[30] Institut für Demoskopie Allensbach (2008) IfD-survey 10016, Allensbach. 10.1016/j.envpol.2015.01.010 25622297

[31] Fershtman C, Gneezy U (2001) Discrimination in a segmented society: An experimental approach. Quarterly Journal of Economics: 351–377.

[32] Purschke C (2011) Regionalsprache und Hörerurteil Grundzüge einer perzeptiven Variationslinguistik. Stuttgart: Steiner 347, XVI S p.

[33] Herrgen J, Schmidt JE (1989) Dialektalitätsareale und Dialektabbau In: Putschke W, Veith WH, Wiesinger P, editors. Dialektgeographie und Dialektologie. Günter Bellmann zum 60. Geburtstag von seinen Schülern und Freunden. Marburg: Elwert pp. 304–346.

[34] Lambert WE, Hodgson RC, Gardner RC, Fillenbaum S (1960) Evaluational Reactions to Spoken Language. Journal of Abnormal and Social Psychology 60: 44–51. 14413611 10.1037/h0044430

[35] Lameli A (2004) Standard und Substandard Regionalismen im diachronen Längsschnitt. Stuttgart: Steiner 272 S p. 10.1016/j.watres.2015.01.006

[36] Schmitt EH (1992) Interdialektale Verstehbarkeit Eine Untersuchung im Rhein- und Moselfränkischen. Stuttgart: Steiner.

[37] Greiner B (2004) An online recruitment system for economic experiments. MPRA Paper No. 13513. Available: http://mpra.ub.uni-muenchen.de/13513/. Accessed: 2014 Feb 14.

[38] Zizzo DJ (2010) Experimenter demand effects in economic experiments. Experimental economics: a journal of the Economic Science Association 13 (1): 75–98.

[39] Fischbacher U (2007) z-Tree. Zurich toolbox for ready-made economic experiments. Experimental Economics 10 (2): 171–178.

[40] Hanushek EA, Schwerdt G, Wiederhold S, Woessmann L (2013) Returns to Skills around the World: Evidence from PIAAC.

[41] Niederle M, Vesterlund L (2007) Do women shy away from competition? Do men compete too much. The Quarterly Journal of Economics 122: 1067–1101.

[42] Grosse ND, Riener G (2010) Explaining gender differences in competitiveness. Gender-task stereotypes. Available: http://zs.thulb.uni-jena.de/receive/jportal_jparticle_00165582. Accessed: 2014 Nov 8

[43] Holt CA, Laury SK (2002) Risk aversion and incentive effects. The American Economic Review 92: 1644–1655.

[44] Durante R, Putterman L, Van der Weele J (2014) Preferences for redistribution and perception of fairness: An experimental study. Journal of the European Economic Associations. 25554727

[45] Huesmann A (1998) Zwischen Dialekt und Standard Empirische Untersuchung zur Soziolinguistik des Varietätenspektrums im Deutschen. Tübingen: Niemeyer XV, 287 S p.

[46] Eckert P (2012) Three Waves of Variation Study: The Emergence of Meaning in the Study of Sociolinguistic Variation. Annual Review of Anthropology 41: 87–100.

[47] Kruger J, Dunning D (1999) Unskilled and unaware of it: How difficulties in recognizing one’s own incompetence lead to inflated self-assessments. Journal of Personality and Social Psychology 77 (6): 1121–1134. 10626367 10.1037//0022-3514.77.6.1121

[48] Wheeler ME, Fiske ST (2005) Controlling racial prejudice and stereotyping: Social cognitive goals affect amygdala and stereotype activation. Psychological Science 16: 56–63. 15660852 10.1111/j.0956-7976.2005.00780.x

