# Correction: The Effect of Perceived Regional Accents on Individual Economic Behavior: A Lab Experiment on Linguistic Performance, Cognitive Ratings and Economic Decisions

**DOI:** 10.1371/journal.pone.0124732

**Published:** 2015-05-13

**Authors:** Stephan Heblich, Alfred Lameli, Gerhard Riener

The order of Figs 1, 2, 3 and 4 is incorrect. Fig 1 should be Fig 3, Fig 2 should be Fig 1, Fig 3 should be Fig 4, and Fig 4 should be Fig 5.

Please see the complete, corrected Fig 1 here.

**Fig 1:**
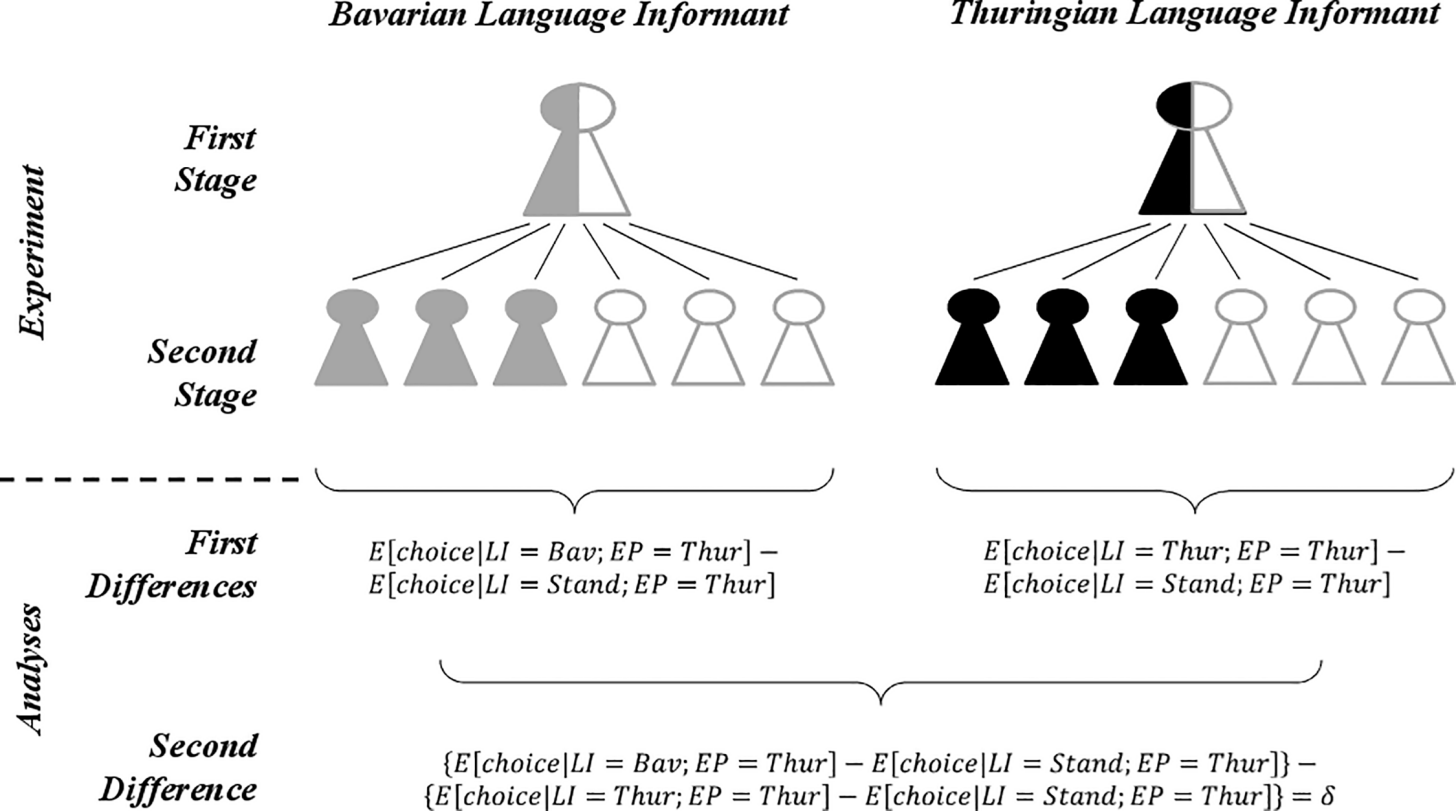
Regional intensity across the language samples. The figure shows the regional intensity of our language samples relative to codified standard German (d = 0). We find a strong and comparable deviation of both regional language samples from codified standard. At the same time, we find an insignificant difference between these two regional accent samples and between the two standard accent samples.

Please see the complete, corrected Fig 2 here.

**Fig 2:**
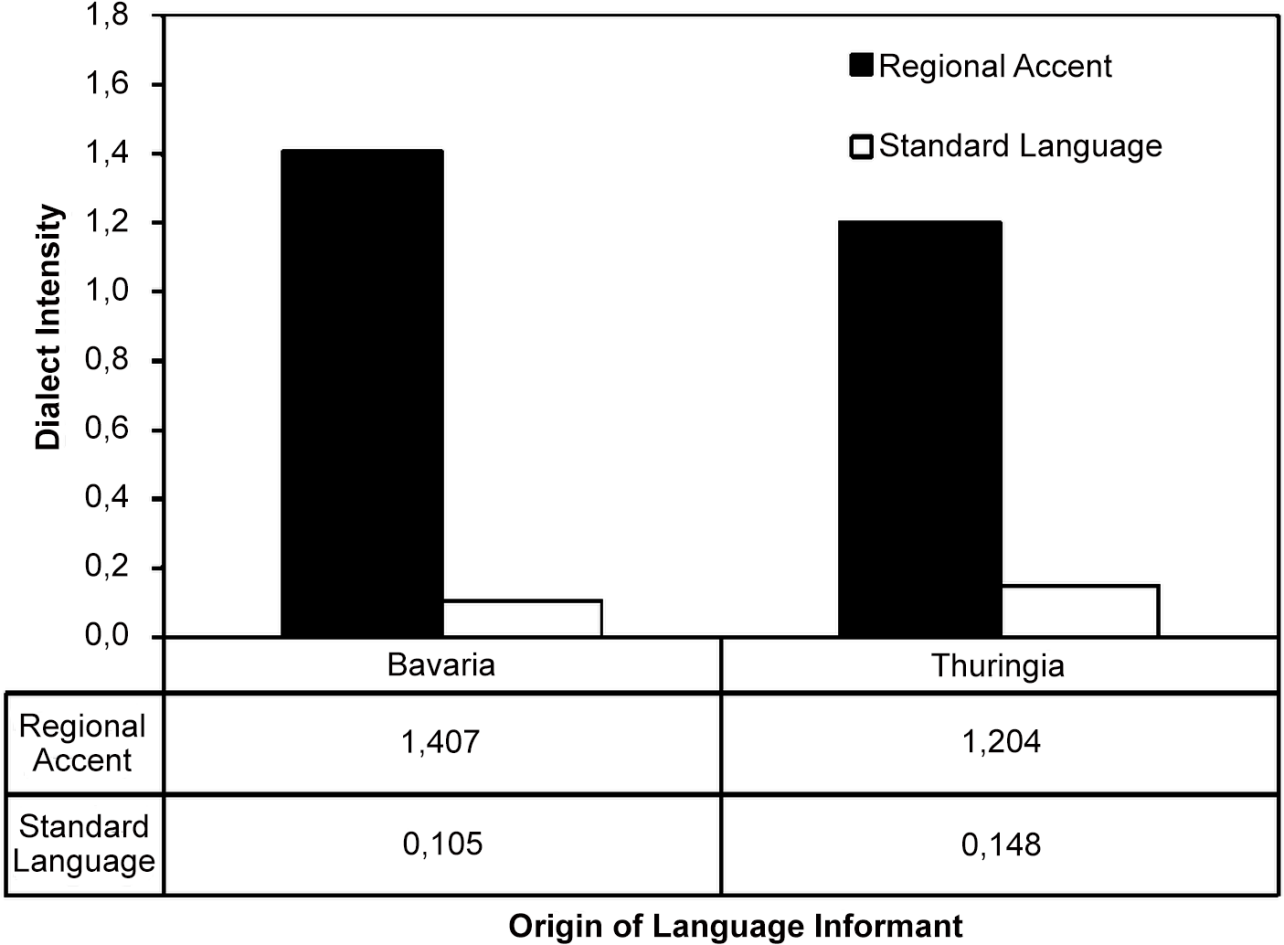
Multi-dimensional plot resulting from the exploration of linguistic loyalty using the questionnaire from (29). Each of the statements below (English translation) was rated on a seven step scale between the poles “completely agree” and “strongly disagree”. Black circles (cluster 1) = statements on dialects with negative connotations (e.g., “Dialect is vulgar.”), black triangles (cluster 4) = statements on dialects with positive connotations (e.g., “Dialect conveys a feeling of security.”), white triangles (cluster 2) = statements on standard German with positive connotations (e.g., “Standard German sounds elegant.”), white circles (cluster 3) = statements on standard German with negative connotations (e.g., “Standard German sounds stiff.”). Kruskal’s test =. 081

Please see the complete, corrected Fig 3 here.

**Fig 3:**
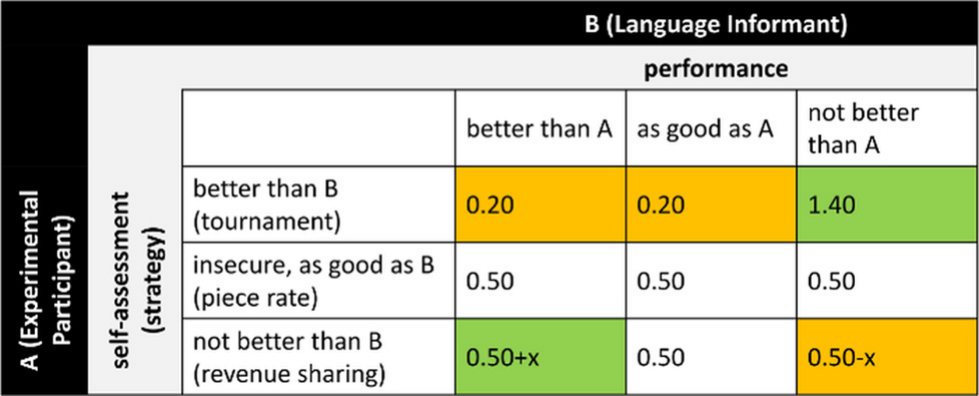
Experimental strategy and subsequent empirical analysis. The standard accent sample (Stand) is shown in white, the Bavarian accent (Bav) in gray, and the Thuringian accent (Thur) in black. The first stage of the experiment shows the two language informants (LI) who provide two language samples each. In the second stage, we relate economically relevant choices to the assigned treatments and match one of four language samples randomly with experimental participants (EP). In the analysis, we first estimate within-speaker differences to eliminate the effect of individual confounding characteristics (First Differences) and then calculate the difference in those first differences (Second Difference) to account for stochastic discrimination against regional accent. Contrasting the expected choices leaves us with an unbiased discrimination effect δ (cf. following explanations).

Please see the complete, corrected Fig 4 here.

**Fig 4:**
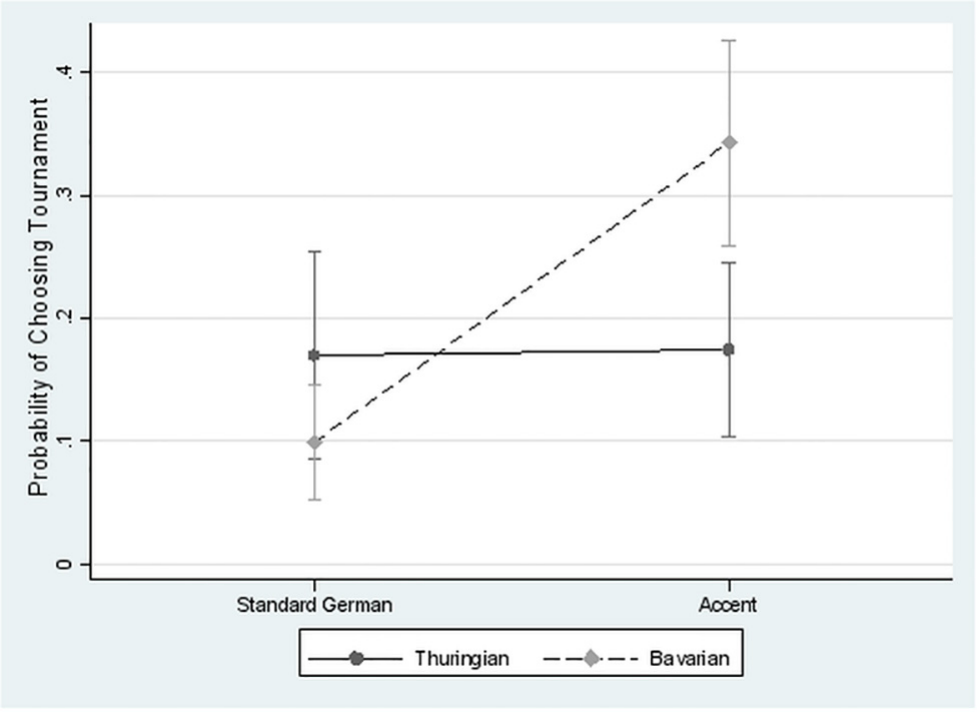
Payoff matrix for tasks 2–5. Color indicates potential gains (green) and losses (orange) compared to piece rate. Piece rate: m = Ʃca * € 0.50; revenue sharing: m = (Ʃca + Ʃcb) / 2 * € 0.50; tournament: m = Ʃca * € 1.40 if Ʃca > Ʃcb otherwise m = Ʃca * € 0.20, with m = payoff; ca = successful completion of task by participant A; cb = successful completion of task by speaker B.

Please see the complete, corrected Fig 5 here.

**Fig 5:**
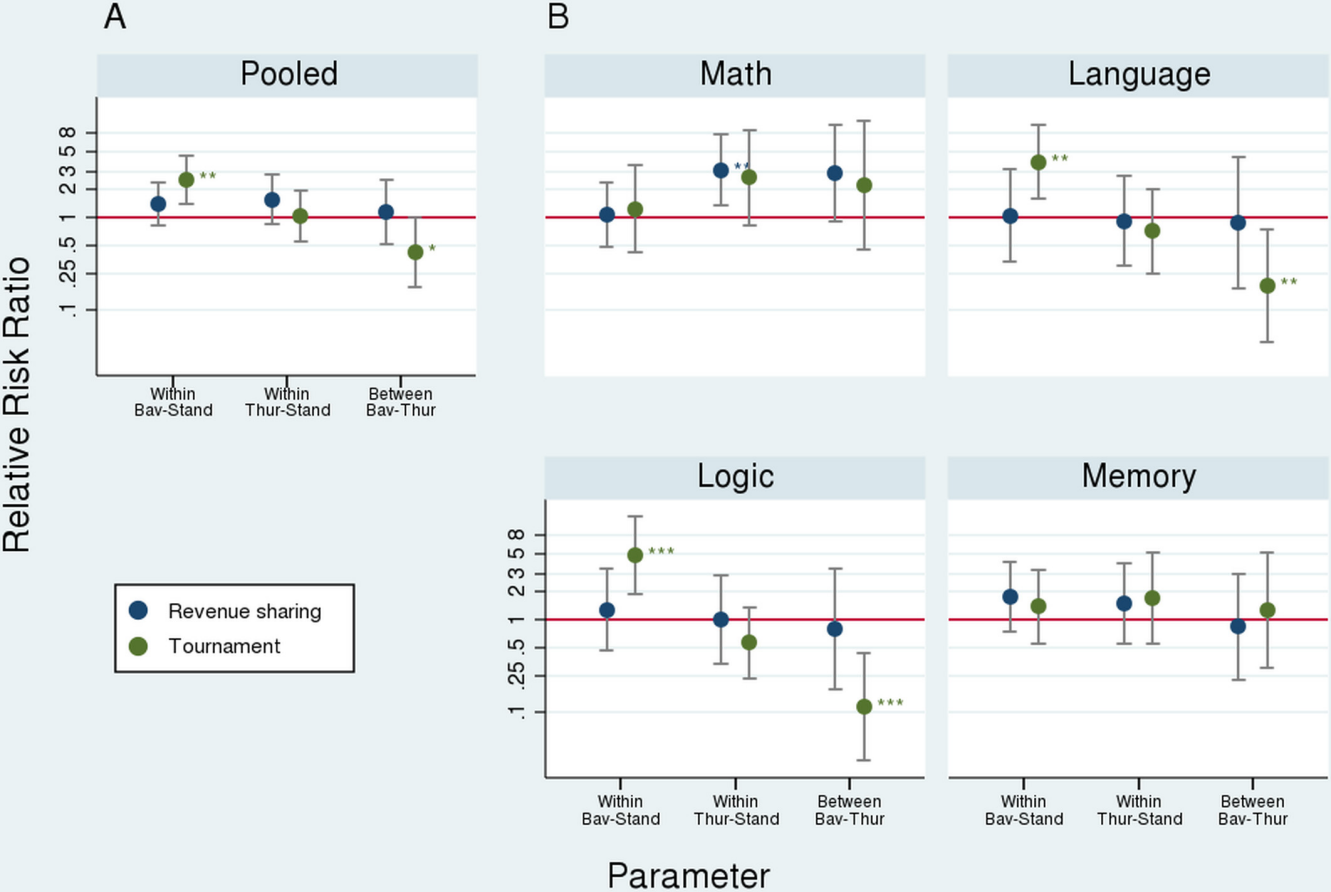
Relative risk ratios (/rr/; log scaled). /Pooled/ refers to the joint results for all tasks using clustered standard errors at the individual level; the remaining graphs present results per task. /Within/ refers to the Analyses—First Differences stage in Fig 1[1] (within-speaker differences) and /Between/ to the Analyses—Second Difference stage (differences between speakers). A rr of one (e.g., Within Bav-Stand; Tournament) would indicate an equal likelihood that EPs choose tournament whether listening to Bavarian accent (/Bav/) or standard accent (/Stand/) while, e.g., a rr of 2.46 would indicate that EPs were 2.46 times more likely to choose tournament when listening to the Bavarian accent than when the same LI were to speak standard accent; A rr of less than one (e.g., Within Thur-Stand; Tournament) indicates that EPs avoid tournament when listening to the Thuringian accent (/Thur/) more often than in the standard accent treatment (/Stand/).

Fig A in Supporting Information file S1_Supporting_Information should be Fig 2.

Fig B in Supporting Information file S1_Supporting_Information should be Fig. A in Supporting Information file S1_Supporting_Information.

The legend for Fig B in S1_Supporting_Information should be deleted. Please view the correct S1_Supporting_Information below.

## Supporting Information

S1 Supporting Information Supporting Information file. Dialect intensity across the speech samples. Fig A shows the results of a formal linguistic test comparing the dialect intensity (i.e. regional accent in the given case) of our speech samples relative to standard language. The figure shows a strong and comparable deviation of both regional speech samples from standard language; the standard German samples are comparable with almost no indication of dialect features. The latter nicely illustrates the dual competence of our language informants. At the same time, we measure a small and insignificant difference in the dialect intensity between the two regional accent samples and between the two standard language samples suggesting that the difference does not influence language perception or affect the semantics of the text (32, 33). We calculate distance as number of micro-phonetic features like voicing, manner, or location of articulation that deviate from standard language divided by the overall number of words in the text (22). A value of d = 0 would suggest perfect compliance with standard language; a value of d = 1 means that, on average, one phonetically feature per word differs from standard language; very pronounced local dialects may have a score of d > 2 or even d > 3 (22, 32, 33). The difference between the regional accent samples is significant at p <. 001, the difference between the samples of the spoken standard language is not significant (cf. main text). Table A: The table shows panel regressions of tournament take-up where the outcome categories of revenue sharing and piece rate are pooled. Column 1 presents the results from a random effects panel regression on the choice of tournament and column 2–4 present mixed models. In column 3 we additionally control for the guessed rank of the EPs. Finally, in column 4 we add a full set of controls. Standard errors in parentheses; *p <. 10, **p <. 05, ***p <. 001. Table B: Linguistic loyalty and payment regime choice: Splitting the sample by loyalty measures, we observe that EPs with a high dialect loyalty (Panel A) choose tournament significantly more often when perceiving the distant Bavarian accent and significantly less often when perceiving the Thuringian accent. The same holds for EPs with standard German loyalty (Panel B), though the effect is less pronounced than it is in the case of dialect loyalty. For those EPs who have a low loyalty for dialects or standard German we do not find any effects. We report relative risk ratios; t statistics in parentheses. *p <. 05, **p <. 01 ***p <. 001
(DOCX)

## References

[1] Heblich S, Lameli A, Riener G (2015) The Effect of Perceived Regional Accents on Individual Economic Behavior: A Lab Experiment on Linguistic Performance, Cognitive Ratings and Economic Decisions. PLoS ONE 10(2): e0113475 doi: 10.1371/journal.pone.0113475 25671607 10.1371/journal.pone.0113475 PMC4324847

