# Supplementary material for: Correction: The Effect of Perceived Regional Accents on Individual Economic Behavior: A Lab Experiment on Linguistic Performance, Cognitive Ratings and Economic Decisions
Source: PLoS One. 2015 May 13;10(5):e0124732. doi: 10.1371/journal.pone.0124732 (PMC4430290; doi:10.1371/journal.pone.0124732)
Supplement: S1 Supporting Information — Dialect intensity across the speech samples. Fig A shows the results of a formal linguistic test comparing the dialect intensity (i.e. regional accent in the given case) of our speech samples relative to standard language. The figure shows a strong and comparable deviation of both regional speech samples from standard language; the standard German samples are comparable with almost no indication of dialect features. The latter nicely illustrates the dual competence of our language informants. At the same time, we measure a small and insignificant difference in the dialect intensity between the two regional accent samples and between the two standard language samples suggesting that the difference does not influence language perception or affect the semantics of the text (32, 33). We calculate distance as number of micro-phonetic features like voicing, manner, or location of articulation that deviate from standard language divided by the overall number of words in the text (22). A value of d = 0 would suggest perfect compliance with standard language; a value of d = 1 means that, on average, one phonetically feature per word differs from standard language; very pronounced local dialects may have a score of d > 2 or even d > 3 (22, 32, 33). The difference between the regional accent samples is significant at p <. 001, the difference between the samples of the spoken standard language is not significant (cf. main text). Table A: The table shows panel regressions of tournament take-up where the outcome categories of revenue sharing and piece rate are pooled. Column 1 presents the results from a random effects panel regression on the choice of tournament and column 2–4 present mixed models. In column 3 we additionally control for the guessed rank of the EPs. Finally, in column 4 we add a full set of controls. Standard errors in parentheses; *p <. 10, **p <. 05, ***p <. 001. Table B: Linguistic loyalty and payment regime choice: Splitting the sample by loyalty measures, w [file pone.0124732.s001.docx]

The Effect of Perceived Regional Accents on Individual Economic Behavior: A Lab Experiment on Linguistic Performance, Cognitive Ratings and Economic Decisions

**Supporting Information A: Material and methods**

In the following we provide texts and examples of the exercises used in tasks 1–5 which are the basis for the payment regimes.

***Listening Comprehension (task 1)***

LKW landet im Graben. Am frühen Mittwochmorgen gegen fünf Uhr sind zwei Lastzüge hintereinander auf der Autobahn unterwegs. Auf einmal schert der hintere LKW, der mit Lebensmitteln beladen ist, aus und setzt zum Überholen an. Als der vordere LKW bremsen muss, gerät der Überholende ins Schleudern und landet im Straßengraben. Der Fahrer wird in seinem Lastzug eingeklemmt. Kurz darauf trifft die Polizei und später die Feuerwehr am Unfallort ein. Auch ein Rettungswagen mit Notarzt wird gerufen, aber der LKW-Fahrer ist schon unverletzt geborgen.

*English Translation*

Truck ends up in the ditch. Early Wednesday morning at five clock two trucks are driving one after another down the highway. Suddenly, the rear truck loaded with supplies changes lane and sets to overtake. As the front truck has to brake, the overtaking truck started to spin and landed in the ditch. The driver is caught in his truck. Shortly thereafter, the police and later the fire brigade arrives at the scene. Also, an ambulance is called, but the truck driver is already recovered unharmed.

***Mathematics (task 2; example)***

| Number 1 | Number 2 | Number 3 | Number 4 | Number 5 | Result |
| --- | --- | --- | --- | --- | --- |
| 22 | 57 | 33 | 78 | 33 | ______ |

***Logic (task 3; example)***

Ein Angestellter bekommt die Aufgabe, sechs Mitarbeitern Büros zuzuteilen. Die Büros sind mit 1-6 nummeriert. Die Büros sind in einer Reihe und nur durch zwei Meter hohe Trennwände geteilt, deswegen kann man hören, was der Nachbar sagt und Zigarettenrauch kann leicht von einem Büro zum anderen gelangen. Frau Roberts benutzt das Telefon sehr oft während des Arbeitstages. Herr Mike und Herr Brown brauchen benachbarte Büros, da sie zusammenarbeiten. Frau Hardy, die Diensälteste, bekommt das Büro mit der Nummer 5, da es das größte Fenster hat. Herr Donald benötigt Ruhe in den benachbarten Büros. Herr Tim, Herr Mike und Herr Donald sind Raucher. Frau Hardy ist allergisch auf Zigarettenrauch und benötigt deshalb ein Büro das von Nichtraucherbüros umgeben ist. Wenn nicht anders angegeben, verhalten sich alle Mitarbeiter ruhig während der Arbeit.

Das ideale Büro für Herrn Mike wäre:

Büro 2 Büro 6 Büro 1 Büro 3 Büro 4

*English Translation*

An employee gets the job to allocate offices to six employees. The offices are numbered 1-6. The offices are divided only by two meter high walls, so you can hear what the neighbor says, and cigarette smoke can easily move from one office to another. Mrs. Roberts uses the phone very often during the workday. Mr. Mike and Mr. Brown need adjacent offices as they work together. Mrs. Hardy, the office eldest, gets the office with the number 5, as it has the largest window. Mr. Donald needs silence in the neighboring offices. Mr. Tim Mr. Mike and Mr. Donald are smokers. Mrs. Hardy is allergic to cigarette smoke and therefore needs an office surrounded by non-smoking offices. Unless otherwise stated, all employees remain quiet while working.

The ideal office for Mr. Mike would be:

Office 2 Office 6 Office 1 Office 3 Office 4

Solution: 4

***Language (task 4; example)***

| Word 1 | Word 2 | Word 3 | Word 4 | Word 5 |
| --- | --- | --- | --- | --- |
| werde | ich | nach Hause | gehen | heute abend |
| Correct Order | |  |  |  |
| 2 | 1 | 4 | 5 | 3 |

*English Translation*

| Word 1 | Word 2 | Word 3 | Word 4 | Word 5 |
| --- | --- | --- | --- | --- |
| will | I | home | go | tonight |

***Memory (task 5)***

Unterhose, Schuhlöffel, Brotmesser, Drahtschere, Fliegengitter, Telefon, Schlüsselanhänger, Bärenfell, Büchergestell, Grubenlampe, Fenstergriff, Feile, Weinglas, Stein, Gleitschirm, Dachpappe

*English Translation*

Underpants, shoehorn, bread knife wire cutters, fly screens phone, key chain, bearskin, book case miner's lamp, window handle, file, wine glass, stone, hang, roofing felt

**Supporting Information B: Loyalty Measure**

The difference-in-differences estimates suggest that EPs chose tournament significantly more often when matched with an out-group regional accent. For a better understanding of the underlying mechanisms driving the observed behavior we turn to the questionnaire that EPs completed at the end of the experiment.

Our questionnaire includes a set of items that provide further insight into the EP’s general linguistic loyalty using validated scales [45] for 36 statements regarding the use of regional varieties in every-day contexts. A terminological problem is the difference between linguistic notions and the layperson’s expressions for language phenomena. Our pretests indicated that *accent* is not the preferred label for the regional language samples under discussion. Instead, *dialect* was the more commonly used notion. To get unambiguous ratings on both regionally marked speech and regionally unmarked speech we thus used the terms *dialect* (“Dialekt”) vs. *standard German* (“Hochdeutsch”). Additionally we verified the EP’s actual linguistic conceptualizations ex-post by asking how they would refer to the language variety they were confronted with: 72.04% of the EPs who were matched with a regional accent referred to it with a dialect concept (e.g. ‘dialect’ or ‘Bavarian’), whereas only 5.38% referred to a concept of accent (e.g., ‘accentual’, ‘dialectally colored’). *Dialect* vs. *standard German* thus proved to be practicable terms.

Before evaluating the questionnaire in the context of our experiment, we tested the suitability of our loyalty measure by means of principal component analysis and multi-dimensional scaling. The results showed a consistent pattern across the 18 dialect and 18 standard German ratings (cf. Fig. A). We extracted four relevant clusters, with two clusters referring to dialect statements and two to standard German statements, each of them clearly separated by positive or negative connotations. The only exception is the statement “Dialect should be cultivated more at school” (Q_08 in Fig. A), which did not fit with the other ratings of this type. As this statement led to untypical response behavior we excluded it from further analysis. This leaves us with a total of 17 dialect statements and 18 standard German statements as basis for a dialect loyalty index and a standard German loyalty index.

Using clusters 1 and 4, we first derived a dialect loyalty index. Both clusters refer to dialect questions but cluster 1 measures negative attitudes to dialect and cluster 4 positive attitudes. To account for the inverse format when combining the loadings of the two components, we constructed the following dialect loyalty index (LD) with

$$LD=\frac{1}{n}\left(\sum_{i=1}^{n} (m-p_{i})+\sum_{i=1}^{n} q_{i}\right)$$

where *p* refers to the statements with positive connotations and *q* to the statements with negative connotations. *m* is the invariant maximum of the rating scales plus 1 (*m*= 8). With this index it becomes possible to determine each EP’s general dialect loyalty which ranges from 1 to 7.

In contrast the index for loyalty with standard German (LSG, clusters 2 and 3) is calculated using:

$$LSG=\frac{1}{n}\left(\sum_{i=1}^{n} (p_{i})+\sum_{i=1}^{n} (m-q_{i})\right)$$

These measures are expressions of an EP’s general loyalty for dialects (regional varieties respectively) and/or standard German as well as his/her cultural appreciation of these varieties. Loyalty to dialect indicates a language-related feeling of regional identity; loyalty to standard German reflects a supra-regional or even national identity. Note that these measures are not mutually exclusive, i.e. we allow for the possibility that both kinds of identity may apply to the same EP. In our experiment, they should be captured in our first differences as they refer to the discrimination against dialect (or standard) German speakers (Table B).

**Fig. A.**

**
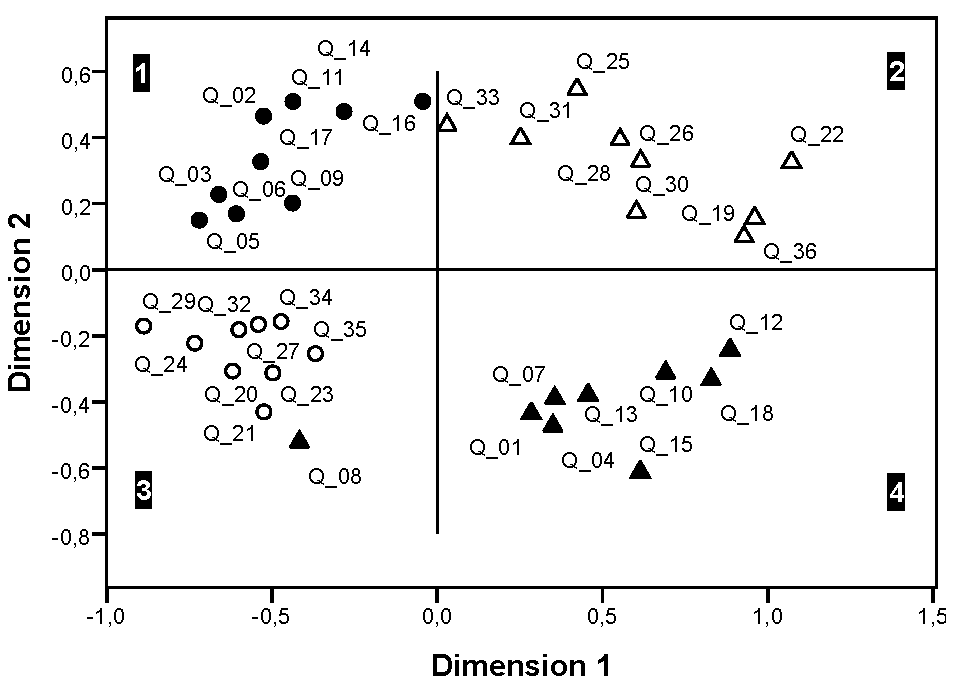
**

| Q_01  Q_02  Q_03  Q_04  Q_05  Q_06  Q_07  Q_08  Q_09  Q_10  Q_11  Q_12  Q_13  Q_14  Q_15  Q_16  Q_17  Q_18 | Dialect conveys a feeling of warmth.  Dialect should increasingly be replaced by standard German.  Dialect is the speech of the lower classes.  Families should accept children speaking dialect.  Dialect is vulgar.  Dialect impedes social progress.  Dialect conveys a feeling of security.  Dialect should be cultivated more at school.  Dialect is repulsive.  Dialect creates a feeling of community.  Dialect should be avoided in my family  Dialect sounds like home.  Dialect is an important part of everyday life  Children should be weaned off dialect in  kindergarten.  Dialects must not be allowed to die out.  Dialect corrupts standard German.  Dialect is ugly.  Dialect preserves the character of our region. |  | Q_19  Q_20  Q_21  Q_22  Q_23  Q_24  Q_25  Q_26  Q_27  Q_28  Q_29  Q_30  Q_31  Q_32  Q_33  Q_34  Q_35  Q_36 | Standard German sounds distinguished.  We have to guard against standard German getting out of hand.  Standard German is very complicated.  Being able to speak standard German is important.  Standard German sounds snobbish.  Standard German gets in the way of personal contact between people.  Standard German is the best German  From kindergarten on, attention should be paid to proper standard German.  Standard German comes across as stand-offish.  Standard German is the foundation of our linguistic culture.  Speaking standard German within a family  should be prevented.  Standard German sounds elegant.  Standard German is the speech of the cultured classes.  It is hard to express your feelings in standard German.  All Germans should speak standard German.  Standard German lacks many ways of expressing oneself in everyday life.  Standard German sounds stiff.  You need standard German to get ahead at work. |
| --- | --- | --- | --- | --- |

Fig. A. Multi-dimensional plot resulting from the exploration of linguistic loyalty using the questionnaire from (*29*). Each of the statements below (English translation) was rated on a seven step scale between the poles “completely agree” and “strongly disagree”. Black circles (cluster 1) = statements on dialects with negative connotations (e.g., “Dialect is vulgar.”), black triangles (cluster 4) = statements on dialects with positive connotations (e.g., “Dialect conveys a feeling of security.”), white triangles (cluster 2) = statements on standard German with positive connotations (e.g., “Standard German sounds elegant.”), white circles (cluster 3) = statements on standard German with negative connotations (e.g., “Standard German sounds stiff.”). Kruskal’s test = .081.

**Table A.**

|  | (1) | (2) | (3) | (4) |
| --- | --- | --- | --- | --- |
|  | Random effects | Mixed model | MM First | MM controls |
| Standard German: Bavarian | -0.045 | -0.045 | -0.045 | -0.012 |
|  | (0.057) | (0.043) | (0.043) | (0.042) |
| Accent: Thuringian | -0.026 | -0.026 | -0.023 | -0.004 |
|  | (0.060) | (0.045) | (0.044) | (0.042) |
| Accent X Bavarian | 0.167** | 0.167*** | 0.179*** | 0.135** |
|  | (0.084) | (0.063) | (0.062) | (0.061) |
| Constant | 0.155** | 0.155** | 0.039 | 0.028 |
|  | (0.045) | (0.039) | (0.037) | (0.109) |
| Logic | 0.066 | 0.066 | 0.164 | 0.115 |
|  | (0.037) | (0.043) | (0.042) | (0.041) |
| Language | 0.144*** | 0.144*** | 0.065 | 0.097** |
|  | (0.042) | (0.046) | (0.050) | (0.047) |
| Memory | 0.024 | 0.024 | -0.052 | -0.017 |
|  | (0.035) | (0.042) | (0.047) | (0.046) |
| Guess: First |  |  | 0.212*** | 0.116*** |
|  |  |  | (0.041) | (0.043) |
| Female |  |  |  | -0.093*** |
|  |  |  |  | (0.034) |
| Envious |  |  |  | 0.136*** |
|  |  |  |  | (0.035) |
| Sharing |  |  |  | 0.036 |
|  |  |  |  | (0.033) |
| Tournament aversion |  |  |  | -0.136*** |
|  |  |  |  | (0.035) |
| Risk aversion | No | No | No | No |
| Observations | 668 | 668 | 668 | 656 |

Table A. Panel regressions of tournament take-up. With the regression analysis presented in this table, we pool the outcome categories of revenue sharing and piece rate. Column 1 presents the results from a random effects panel regression on the choice of tournament. In the aggregate, Thuringian subjects chose tournament more often when they listen to a Bavarian accent. This result remains robust when estimating a mixed model, presented in column 2. The difference also pertains when controlling for the guessed rank of the subjects (column 3). Unsurprisingly, subjects chose the tournament more often when they think they are better than their opponent, but this does not change the size or the significance of the treatment variables. Other controls have the expected signs (presented in column 4): Females and subjects who indicated in a control task that they are tournament averse tend to take competition less often, which is driven by the mathematics and logic task, while envious people are more likely to engage in tournament. However, we do not observe interaction effects (not reported) between the language treatments and gender, indicating that men and women discriminate equally strongly. Across all tasks the results from Fig. 4 are confirmed, tournament take-up is significantly higher when the language informant speaks with a Bavarian accent. This result is driven by two tasks: the logic task and the language task, while in the mathematics task and the memory task we do not observe that behavior. Standard errors in parentheses; *p < .10, **p < .05, ***p < .001

**Table B.**

|  | **Panel A** |  |  |  |  |  |  |  |  |  |
| --- | --- | --- | --- | --- | --- | --- | --- | --- | --- | --- |
|  | (1) |  | (2) |  | (3) |  | (4) |  | (5) |  |
|  | All |  | Math |  | Logic |  | Language |  | Memory |  |
| Dependent variable: Regime | RS | Tourn | RS | Tourn | RS | Tourn | RS | Tourn | RS | Tourn |
| Accent Bavarian | 1.482 | 3.453*** | 1.286 | 2.528 | 0.835 | 3.826* | 1.804 | 5.863** | 2.058 | 2.533 |
|  | (1.15) | (2.98) | (0.48) | (1.16) | (-0.25) | (1.87) | (0.94) | (2.46) | (1.27) | (1.43) |
| Standard German: Thuringian | 0.517 | 1.496 | 0.212*** | 0.377 | 1.143 | 1.905 | 0.719 | 5.750** | 0.516 | 0.496 |
|  | (-1.58) | (0.95) | (-2.61) | (-1.00) | (0.19) | (0.81) | (-0.42) | (2.41) | (-1.01) | (-0.88) |
| Accent X Thuringian | 1.474 | 0.364* | 4.334* | 2.482 | 1.372 | 0.249 | 0.934 | 0.0958** | 0.894 | 0.757 |
|  | (0.72) | (-1.71) | (1.83) | (0.76) | (0.32) | (-1.38) | (-0.07) | (-2.56) | (-0.13) | (-0.27) |
| Constant | 0.443*** | 0.177*** | 1.231 | 0.231** | 0.208*** | 0.125*** | 0.261*** | 0.130*** | 0.421* | 0.263*** |
|  | (-3.10) | (-5.30) | (0.55) | (-2.28) | (-3.18) | (-3.38) | (-2.92) | (-3.31) | (-2.04) | (-2.65) |
| Observations | 536 |  | 134 |  | 134 |  | 134 |  | 134 |  |
|  |  |  |  |  |  |  |  |  |  |  |
|  | **Panel B** |  |  |  |  |  |  |  |  |  |
|  | (1) |  | (2) |  | (3) |  | (4) |  | (5) |  |
|  | RS | Tourn | RS | Tourn | RS | Tourn | RS | Tourn | RS | Tourn |
| Accent Bavarian | 1.026 | 2.125* | 0.911 | 1.429 | 0.500 | 2.667* | 1.158 | 3.474* | 1.228 | 1.349 |
|  | (0.08) | (1.93) | (-0.18) | (0.55) | (-0.90) | (1.73) | (0.24) | (2.05) | (0.38) | (0.50) |
| Standard German: Thuringian | 0.568 | 0.957 | 0.307** | 0.455 | 0.764 | 1.069 | 1.048 | 2.933* | 0.476 | 0.389 |
|  | (-1.47) | (-0.10) | (-2.14) | (-1.12) | (-0.40) | (0.11) | (0.07) | (1.73) | (-1.27) | (-1.34) |
| Accent X Thuringian | 1.564 | 0.445 | 3.802* | 1.027 | 2.504 | 0.224* | 0.818 | 0.182** | 1.018 | 1.390 |
|  | (0.89) | (-1.41) | (1.76) | (0.03) | (0.90) | (-1.67) | (-0.22) | (-2.06) | (0.02) | (0.35) |
| Constant | 0.534** | 0.329*** | 1.333 | 0.500 | 0.273** | 0.273*** | 0.318** | 0.227*** | 0.588 | 0.412** |
|  | (-2.54) | (-3.59) | (0.75) | (-1.38) | (-2.81) | (-2.81) | (-2.63) | (-2.98) | (-1.33) | (-1.97) |
| Observations | 576 |  | 144 |  | 144 |  | 144 |  | 144 |  |

Table B. Linguistic loyalty and payment regime choice: Splitting the sample by loyalty measures, we observe that subjects with a high dialect loyalty (Panel A) choose tournament significantly more often when listening to the distant Bavarian accent and significantly less often when matched with the Thuringian accent. The same holds for subjects with standard German loyalty (Panel B), though the effect is less pronounced than it is in the case of dialect loyalty. For those participants who have a low loyalty for dialects or standard German we do not find any effects. We report relative risk ratios; t statistics in parentheses. *p < .05, **p < .01 ***p < .001
